# Time–frequency characteristics of scalp high‐frequency oscillations provide complementary biomarkers in infantile epileptic spasms syndrome

**DOI:** 10.1113/EP094250

**Published:** 2026-08-21

**Authors:** Shunta Yamaguchi, Keisuke Maeda, Naoko Ishihara, Gen Furukawa, Himari Tsuboi, Keisuke Osakabe, Keiko Sugimoto, Naohiro Ichino

**Affiliations:** ^1^ Department of Clinical Laboratory Sciences, Field of Clinical Laboratory Sciences Fujita Health University Graduate School of Medical Sciences Toyoake Japan; ^2^ Department of Clinical Physiology Fujita Health University School of Medical Sciences Toyoake Japan; ^3^ Department of Pediatrics Fujita Health University School of Medicine Toyoake Japan; ^4^ Department of Clinical Laboratory Fujita Health University Hospital Toyoake Japan; ^5^ Department of Medical Sciences Education Fujita Health University School of Medical Sciences Toyoake Japan

**Keywords:** epileptic spasm, high‐frequency oscillation, hypsarrhythmia, infantile epileptic spasms syndrome, interictal epileptiform discharges

## Abstract

Infantile epileptic spasms syndrome (IESS) is characterized by epileptic spasms (ES) in infants and hypsarrhythmia on EEG. High‐frequency oscillations (HFOs), defined as oscillatory events at >80 Hz, reflect rapid spatiotemporal dynamics of cortical activity and might serve as novel biomarkers for epilepsy. In this study, we investigated the association among hypsarrhythmia, ES and scalp HFOs in IESS, focusing on their spatiotemporal and morphological characteristics. We analysed scalp EEG recordings from 22 paediatric patients with IESS (12 males; age range, 0–12 years), classified into four groups: hypsarrhythmia with ES; hypsarrhythmia without ES; multifocal interictal epileptiform discharges (IED); and non‐multifocal IED. The proportions of EEG recordings with at least one detected scalp HFO differed significantly among the groups, being 100.0% in hypsarrhythmia with ES, 90.9% in hypsarrhythmia without ES, 36.0% in multifocal IED and 29.4% in non‐multifocal IED (*P* < 0.001). Age‐adjusted odds ratios [95% confidence intervals] for high HFO detection rates were significantly greater in the hypsarrhythmia groups than in the IED groups (with ES, 24.66 [4.86–125.10], *P* < 0.001; and without ES, 8.38 [1.94–36.20], *P* = 0.004). Receiver operating characteristic analysis showed that scalp HFO detection rates discriminated ES activity with an area under the curve of 0.92, 100% sensitivity and 77.4% specificity. Compared with the IED groups, the hypsarrhythmia groups exhibited lower‐frequency HFOs, with the lowest HFO frequencies being observed in the hypsarrhythmia with ES group. These findings suggest that the spatiotemporal and morphological characteristics of scalp HFOs might provide complementary multidimensional biomarkers for hypsarrhythmia and assessing ictal states in IESS.

## Abstract (highlights)

- **What is the central question of this study?**
  Scalp high‐frequency oscillations (HFOs) have been reported in infantile epileptic spasms syndrome (IESS). However, whether scalp HFOs are associated with hypsarrhythmia and the presence of epileptic spasms (ES) remains unclear.
- **What is the main finding and its importance?**
  Scalp HFOs were detected more frequently in EEG recordings with hypsarrhythmia. Patients with hypsarrhythmia, particularly those with ES, exhibited distinct HFO characteristics, exhibiting lower frequencies compared with the IED groups, with the lowest frequencies being observed in patients with ES. Detection rates and morphological features of HFOs provide complementary biomarkers for hypsarrhythmia and ictal states in IESS.

## 1 INTRODUCTION

Infantile epileptic spasms syndrome (IESS), a clinical term introduced by the International League Against Epilepsy (ILAE) (Zuberi et al., 2022), is characterized by epileptic spasms (ES) in infants and represents one of the most severe forms of epileptic encephalopathy (Ng et al., 2024; Snyder et al., 2024; Specchio et al., 2022). This syndrome encompasses both West syndrome and cases of infants with ES who do not meet all the diagnostic criteria for West syndrome (Zuberi et al., 2022). It is characterized by ES, which typically occur in clusters and are often observed upon waking, along with a pattern of hypsarrhythmia on interictal EEG and developmental regression or stagnation (Bashiri et al., 2024; Fukuyama, 2001). Long‐term developmental outcomes of IESS are strongly influenced by early diagnosis, prompt initiation of treatment and the chosen therapeutic strategy (Al‐Omari et al., 2025; O'Callaghan et al., 2011). Therefore, tools that support early identification and therapeutic management of IESS might facilitate timely clinical decisions and, ultimately, improve long‐term developmental outcomes.

Hypsarrhythmia is a disorganized EEG pattern characterized by irregular, high‐amplitude slow waves in the <3 Hz frequency range, interspersed with sharp waves and spikes (Janicot et al., 2020). This abnormal EEG pattern is thought to be involved in both ES and developmental regression in West syndrome (Janicot et al., 2020). Moreover, some studies have suggested that the duration of hypsarrhythmia might also influence developmental outcomes (Primec et al., 2006). ES vary substantially not only among patients, ranging from a few episodes per day to >100 episodes daily, but also within the same patient over time, particularly before and after treatment. The presence and frequency of ES, in addition to the underlying epileptic network excitability, cannot be evaluated sufficiently based solely on conventional EEG findings, such as hypsarrhythmia (Smith et al., 2026). Importantly, both the presence and frequency of ES are crucial factors in determining treatment strategies and predicting clinical outcomes. Nevertheless, assessment of ES currently relies heavily on clinical information obtained from patients and caregivers, in addition to prolonged long‐term video‐EEG monitoring, including overnight recordings (Gaily et al., 2001; Smith et al., 2026). The primary therapeutic goal in IESS is to eliminate both ES and interictal hypsarrhythmia (Primec et al., 2006).

High‐frequency oscillations (HFOs) are defined as events with a frequency of >80 Hz, consisting of at least four oscillations, and clearly standing out from the background activity (Jacobs & Zijlmans, 2020). HFOs were initially reported in animal models and later observed in human intracranial EEG, leading to the expectation that HFOs could serve as a new biomarker for epilepsy (Akiyama et al., 2005; Bragin et al., 1999; Jirsch et al., 2006; Maeda, Hosoda, Fukumoto, Kawai et al., 2025, Maeda, Hosoda, Fukumoto, Tsuboi et al., 2025, Maeda, Hosoda, Tsuboi et al., 2025, Maeda, Tsuboi et al., 2025, Maeda, Tsuboi, Hosoda et al., 2025, 2026). Recent studies have shown that HFOs can also be measured using non‐invasive scalp EEG and that scalp‐detected HFOs reflect cortical HFOs (Pizzo et al., 2016). Several previous studies have reported that scalp HFOs are associated with the localization of epileptogenic activity (Menendez de la Prida & Gotman, 2024; Nariai et al., 2011). Additionally, scalp HFOs have been reported to be correlated with spasms, treatment response and disease severity in patients with West syndrome (Maeda, Tsuboi, Hosoda et al., 2025; Menendez de la Prida & Gotman, 2024; Nariai et al., 2011). However, to our knowledge, few studies have investigated not only the relationship between hypsarrhythmia and scalp HFOs, but also, more importantly, the association between ES and scalp HFOs in patients with IESS. Although visual validation remains necessary, scalp HFO detection can be assessed semi‐automatically and objectively (Noorlag et al., 2022; Wong et al., 2021). By clarifying the relationship among hypsarrhythmia, ES and scalp HFOs, the HFOs might serve not only as an objective indicator of hypsarrhythmia, but also as an objective biomarker of ES activity beyond conventional EEG findings.

Given this background, the aim of the present study was to examine the association among hypsarrhythmia, ES and scalp HFOs recorded by scalp EEG in patients with IESS. The findings could be expected to contribute to the establishment of scalp HFOs as an adjunctive tool for the diagnosis and therapeutic management of IESS.

## 2 MATERIALS AND METHODS

### 2.1 Ethical approval

This study was approved by the Ethics Committee of Fujita Health University (approval no. HM22‐143). The requirement for informed consent from patients and their guardians was waived for the following reasons. All clinical information and EEG recordings were depersonalized to ensure patient anonymity. The EEG recordings used in this study were routinely acquired for clinical purposes and were analysed after anonymization; no additional examination or interventions were performed for research purposes. To ensure that patients had the opportunity to opt out, information regarding the study was disclosed on the website of Fujita Health University. This study was conducted in accordance with the principles of the *Declaration of Helsinki* and its subsequent amendments, except for registration in a database. We confirm that the study adhered to the specific ethical guidelines for human experiments outlined in the journal's Information for Authors.

### 2.2 Patients

This study enrolled paediatric patients diagnosed with IESS by a paediatric neurologist according to the ILAE criteria who had undergone routine scalp EEG at Fujita Health University Hospital between March 2018 and May 2024. For each patient, scalp EEG recordings, including recordings obtained from the same patient on different days, were selected based on the following inclusion criteria: (1) sampling frequency of ≥1000 Hz; (2) recorded during intermittent seizures; and (3) containing sufficient non‐rapid eye movement (NREM) sleep. EEG recordings from patients who had received adrenocorticotrophic hormone (ACTH) therapy within the previous 6 months were excluded because a previous study has reported that HFOs decrease markedly following ACTH therapy (Kobayashi et al., 2015). A total of 66 scalp EEG recordings from 22 paediatric patients (12 males) met the inclusion criteria and were included in the final analysis.

All EEG recordings were categorized into the following four groups based on EEG findings and the presence of ES: hypsarrhythmia with ES (*n* = 13); hypsarrhythmia without ES (*n* = 11); multifocal interictal epileptiform discharges (multifocal IED, *n* = 25) not fulfilling the criteria for hypsarrhythmia; and non‐multifocal IED (*n* = 17). These EEG patterns, including hypsarrhythmia, were identified according to the ILAE criteria. In this study, ‘with ES’ was defined as clinically active ES with the presence of at least one ES per day, regardless of whether ictal events were captured during the EEG recording; all other cases were categorized as ‘without ES’.

### 2.3 Scalp EEG recording and data selection

Scalp EEG data were obtained using the Neurofax system (Nihon‐Kohden, Tokyo, Japan) at a sampling frequency of 1000 Hz. EEG recordings were acquired according to the international 10–20 system, and analyses were conducted using the following channels in an average montage: Fp1, Fp2, F3, F4, C3, C4, P3, P4, O1, O2, F7, F8, T3, T4, T5, T6, Fz, Cz and Pz. Approximately 600 s of NREM sleep EEG was selected and visually inspected by a specialist technician certified by the Japanese Society of Clinical Neurophysiology. In this selection process, EEG epochs were chosen specifically to minimize the influence of EMG activity and movement artefacts, with consideration of the simultaneously recorded EMG data (e.g., deltoid EMG). Channels containing significant artefacts were excluded from the analyses. All EEG data were stored in European Data Format.

### 2.4 Automatic and manual detection of scalp HFOs

Detection of scalp HFOs was performed using the HFO App and MATLAB R2022a software (MathWorks, Natick, MA, USA) (Zhou et al., 2021). Scalp HFOs were defined as events with a frequency of >80 Hz, consisting of at least four oscillations, and clearly distinguishable from background activity (Jacobs & Zijlmans, 2020). Automated detection was conducted using the Hilbert detector implemented by Crépon et al. (2010). Raw EEG signals were bandpass filtered between 80 and 250 Hz using a linear‐phase finite impulse response filter implemented in MATLAB, as provided in the HFO App (Zhou et al., 2021). The signal envelope was computed using the Hilbert transform, and local maxima were detected automatically using a threshold set to 5 SD of the envelope calculated over the entire recording. All automatically detected scalp HFOs were subsequently validated through visual inspection. For this purpose, time–frequency spectrograms were calculated using a continuous Gabor wavelet transform (Navarrete et al., 2016). Candidate scalp HFO events detected by the Hilbert detector were displayed with the event centred within the analysis window, allowing visual inspection of distinct frequency peaks while minimizing boundary effects associated with the wavelet transform. This facilitated the discrimination of genuine scalp HFOs from broadband artefacts. Specifically, any high‐frequency signals that exhibited broad‐spectrum noise or lacked a circumscribed power peak in the time–frequency analysis were rigorously excluded (Bénar et al., 2010). Visual verification was performed by a specialist technician certified by the Japanese Society of Clinical Neurophysiology. The scalp HFO detection rate (detections per minute) was calculated by dividing the total number of detected scalp HFO events across all channels by the duration of the EEG analysed.

### 2.5 HFO characterization

HFO characteristics were quantified by calculating the frequency, duration, amplitude (peak *z*‐score) and number of cycles for all detected scalp HFOs for each patient. Among all detected HFOs (*n* = 865), HFOs with overlapping time windows across multiple EEG channels were regarded as simultaneously detected. Such events were considered likely to represent the same underlying event rather than independent observations. Therefore, when HFOs were detected concurrently in multiple EEG channels, only the event with the largest amplitude was retained as the representative HFO for subsequent analysis, and the remaining simultaneously detected HFOs were excluded. After applying this criterion, 572 HFO events were included in the final analysis. Frequency was calculated based on crest‐to‐trough intervals of the oscillatory waveform. Duration was defined as the time during which the Hilbert envelope of the signal remained above the detection threshold. Amplitude (peak *z*‐score) was calculated as the maximum peak‐to‐peak value within the event and expressed as a *z*‐score normalized to the baseline period. The number of cycles was estimated by dividing the total event duration by the average interval between consecutive peaks.

### 2.6 Automatic and manual detection of spikes

For the purposes of this study, all epileptiform discharges, including spikes and sharp waves, were referred to collectively as a ‘spike’. The presence or absence of spikes in each EEG recording was determined initially by clinical epilepsy specialists. Only EEG recordings in which spikes were identified visually were subsequently analysed using the Persyst 14 Spike Detector (Persyst Development Corporation, San Diego, CA, USA), one of the most widely used automated spike detection algorithms in clinical EEG analysis. Previous large‐scale validation studies have demonstrated that its detection performance is comparable to expert visual EEG interpretation (Joshi et al., 2018; Scheuer et al., 2017). The spike detection rate (detections per minute) was calculated by dividing the total number of automatically detected spike events across all channels by the duration of the EEG analysed.

### 2.7 Data collection

The diagnosis of IESS was made in accordance with the 2017 ILAE criteria by clinical epilepsy specialists who were blinded to the HFO analysis. In this study, patients were included if the initial onset of epileptic spasms occurred between 1 and 24 months of age, consistent with the diagnostic age window for IESS. Some EEG recordings were obtained after 24 months of age because the cohort included patients with clinical relapse, persistent disease activity or evolution into Lennox–Gastaut syndrome (*n* = 4) at the time of EEG recording. Clinical information, including sex, age at the time of the EEG recording, age at IESS onset and medication history, was obtained through a retrospective chart review.

### 2.8 Statistical analysis

Continuous variables with a normal distribution were presented as means ± SDs, whereas non‐normally distributed variables, such as the scalp HFO detection rate and age at the time of the EEG recording, were presented as medians with interquartile ranges (IQRs). To account for the non‐independence of repeated EEG recordings within patients (66 EEG recordings from 22 patients), analyses were performed using statistical models that accounted for the hierarchical structure of the data. Specifically, the proportion of EEG recordings exhibiting scalp HFOs (categorical variable) was compared among the four groups [hypsarrhythmia with ES (*n* = 13); hypsarrhythmia without ES (*n* = 11); multifocal IED (*n* = 25); and non‐multifocal IED (*n* = 17)] using a generalized estimating equation (GEE) model with a logit link function and an exchangeable correlation structure, with patient identity (ID) specified as the clustering variable. The overall group effect was evaluated using the Wald test. Likewise, the scalp HFO detection rate (continuous variable) was compared among the four groups using a linear mixed‐effects model (LMM), with patient ID included as a random effect. EEG recordings with scalp HFOs were divided further into high‐ and low‐detection rate groups based on the median detection rate (0.22 detections/min). A generalized linear mixed model (GLMM) with a logit link was conducted to estimate odds ratios (ORs) and 95% confidence intervals (CIs) for the high scalp HFO detection rate among the hypsarrhythmia with ES, hypsarrhythmia without ES and IED (multifocal, non‐multifocal) groups, with patient ID included as a random effect. Receiver operating characteristic (ROC) curve analysis was performed to evaluate and compare abilities of scalp HFO detection rates and spike detection rates to discriminate the presence of ES, defined as distinguishing the hypsarrhythmia with ES group from the remaining three groups (hypsarrhythmia without ES, multifocal IED and non‐multifocal IED). To account for repeated EEG recordings obtained from the same patient, GLMMs with a binomial distribution and logit link function were fitted for each predictor, with patient ID included as a random intercept. Predicted probabilities derived from the GLMMs were used to construct ROC curves. Sensitivity, specificity and the area under the ROC curve (AUC) were calculated. To obtain robust estimates of the AUC and 95% CIs, cluster bootstrap resampling was performed with 1000 iterations, with resampling conducted at the patient level to preserve the within‐patient correlation structure. Additionally, scalp HFO characteristics were analysed at the event level (572 scalp HFOs from 66 EEG recordings). Four features (frequency, amplitude, duration and number of cycles) were compared among hypsarrhythmia with ES (*n* = 307), hypsarrhythmia without ES (*n* = 157) and IED groups (*n* = 108) using nested LMMs, with both patient ID and EEG recording date included as random effects to account for the nested data structure. Given that frequency, amplitude and the number of cycles followed a log‐normal distribution, log‐transformed values were used for the analysis. Trend analyses were performed to evaluate the linear associations across the three categories (from IED to hypsarrhythmia without ES, then to hypsarrhythmia with ES). Furthermore, LMMs were performed to estimate unstandardized beta coefficients (β), standardized beta coefficients (standardized β) and 95% CIs for differences among the hypsarrhythmia with ES, hypsarrhythmia without ES and IED groups. These analyses were adjusted for age at the time of the EEG recording. Statistical significance was set at *P *< 0.05. All statistical analyses were performed using Python (statsmodels, scipy, numpy), R (v.4.6.1) and JMP software (v.18; SAS Institute, Inc., Cary, NC, USA).

## 3 RESULTS

### 3.1 Basic characteristics

Table 1 shows the basic characteristics of the study participants. A total of 22 paediatric patients with IESS [12 males (54.5%) and 10 females (45.5%)] were included in the final analysis. The median [IQR] age at the time of EEG recording was 2.3 [0.7–5.3] years. Detailed information on all EEG recordings is provided in Appendix Table A1.

**TABLE 1:** Basic characteristics of the 22 paediatric patients diagnosed with infantile epileptic spasms syndrome.

Characteristic	Patients with IESS (*n* = 22)
Sex, *n*, (%) Male Female	12 (54.5) 10 (45.5)
Age at the time of the EEG recording, years^a^	2.3 (0.7–5.3)
Age at the time of the first seizure, months^a^	5.0 (3.5–7.0)
Duration since the last seizure, days^a^	1.3 (0.6–31.2)
Age at IESS onset, years^a^	0.0 (0.0–0.0)
IESS duration, days^a^	587.5 (46.6–1820.4)
Medication user, *n* (%)	
Valproic acid	10 (45.5)
Vigabatrin	6 (27.2)
Zonisamide	5 (22.7)
Levetiracetam	1 (4.5)
Perampanel	1 (4.5)
Topiramate	1 (4.5)
Others	8 (36.4)
Two drugs combined	5 (22.7)
More than two drugs combined	6 (26.1)
Analysed EEG recording duration, s^a^	601.0 (563.8–601.0)
Sleep‐including agent use during EEG recording, *n* (%)	10 (45.5)

Abbreviation: IESS, infantile epileptic spasms syndrome.

^a^ Data are presented as the median and interquartile range (25th–75th percentiles).

### 3.2 Comparison of scalp HFOs among the four groups: hypsarrhythmia with ES; hypsarrhythmia without ES; multifocal IED; and non‐multifocal IED

Figure 1a,e shows EEG recordings from patients in the IED and hypsarrhythmia groups, displayed in unfiltered conditions with standard time scales. Figure 1b,f shows unfiltered EEG recordings with expanded time scales enclosed in red rectangles, and Figure 1c,g shows bandpass filtering at 80–250 Hz. Figure 1d,h shows spectrograms of the EEG data in a time–frequency analysis for the recordings enclosed in red rectangles. A single scalp HFO was observed in the EEG recording of IED, whereas numerous scalp HFOs were observed in the EEG recording of hypsarrhythmia. To provide an overview of the spectral characteristics of the entire EEG, a representative broadband power spectrum of the 600 s EEG recording used for analysis is provided in Appendix Figure A1.

**FIGURE 1:**
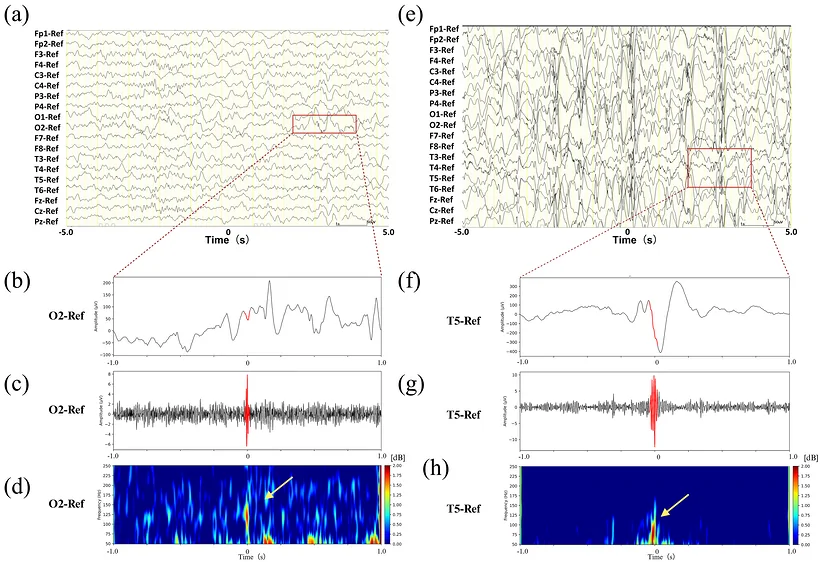
Representative scalp HFOs detected in patients with IESS. (a–d) EEG recordings of IED. (e–h) EEG recordings of hypsarrhythmia. (a, e) Unfiltered EEG recordings with standard time scales. (b, f) Unfiltered EEG recordings with expanded time scales enclosed within red rectangles. (c, g) Bandpass‐filtered EEG (80–250 Hz) with expanded time scales enclosed within red rectangles. (d, h) Spectrograms of the EEG data in the time–frequency analysis for the recordings enclosed within red rectangles. The colormap unit represents spectral power (expressed in decibels) relative to the mean power during the artefact‐free baseline period (0.0–0.2 s in the epoch). Scalp HFOs are visible in the unfiltered and filtered EEG recordings (red traces) and correspond to spectral blobs in the time–frequency analysis (yellow arrows). Abbreviations: HFO, high‐frequency oscillation; IED, interictal epileptiform discharge; IESS, infantile epileptic spasms syndrome.

Figure 2a presents a mosaic plot showing the proportion of EEG recordings with at least one scalp HFO detected across the four groups. The raw proportions were 100.0% in the hypsarrhythmia with ES group, 90.9% in the hypsarrhythmia without ES group, 36.0% in the multifocal IED group and 29.4% in the non‐multifocal IED group. The analysis using a GEE model confirmed that these proportions differed significantly among the four groups (Wald test, *P *< 0.001).

**FIGURE 2:**
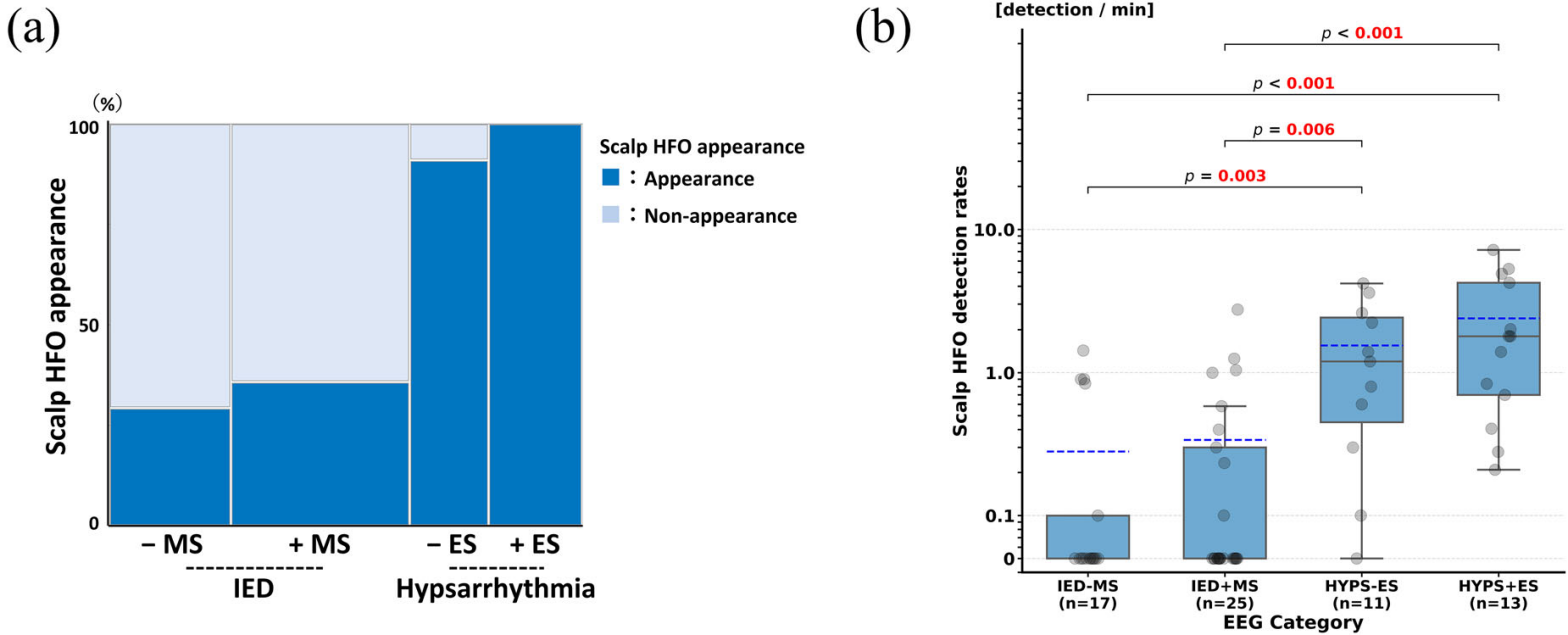
Comparison of scalp HFOs among the four groups: hypsarrhythmia with ES; hypsarrhythmia without ES; multifocal IED; and non‐multifocal IED. (a) Mosaic plot showing the proportion of EEG recordings with at least one scalp HFO detected in each group. (b) Box plot comparing scalp HFO detection rates among the four groups. Each box plot shows the median (bold horizontal line), mean (dotted horizontal line), interquartile range (box) and non‐outlier range (whiskers). *P*‐values were calculated using a linear mixed‐effects model. Abbreviations: ES, epileptic spasms; HFO, high‐frequency oscillation; IED, interictal epileptiform discharge; IESS, infantile epileptic spasms syndrome; MS, multifocal spike.

Figure 2b presents a box plot comparing the scalp HFO detection rates among the four groups using an LMM. The median detection rate was significantly higher in the hypsarrhythmia with ES group (median [10th–90th]: 1.8 [0.2–6.4]) than in the multifocal IED group (0.0 [0.0–1.1], both crude and age‐adjusted *P *< 0.001) and the non‐multifocal IED group (0.0 [0.0–1.0], both crude and age‐adjusted *P *< 0.001). Likewise, the hypsarrhythmia without ES group (1.2 [0.0–4.1]) showed a significantly higher detection rate than both the multifocal and non‐multifocal IED groups (multifocal IED, crude *P *= 0.007, age‐adjusted *P *= 0.006; and non‐multifocal IED, crude *P *= 0.006, age‐adjusted *P *= 0.003).

### 3.3 ORs and 95% CIs of high scalp HFO detection rates in EEG recordings with hypsarrhythmia with and without ES relative to IED

Table 2 shows the ORs and 95% CIs of high scalp HFO detection rates in EEG recordings with hypsarrhythmia with and without ES groups relative to the IED (multifocal, non‐multifocal) group using a GEE model. The crude ORs [95% CIs] for high detection rates were significantly higher in EEG recordings with hypsarrhythmia with and without ES compared with those with IED (with ES, 17.13 [3.47–84.70], *P *< 0.001; without ES, 7.38 [1.75–31.06], *P *= 0.006). After adjusting for age at the time of the EEG recording, the ORs remained significantly higher (with ES, 24.66 [4.86–125.10], *P *< 0.001; without ES, 8.38 [1.94–36.20], *P *= 0.004).

**TABLE 2:** Odds ratios and 95% confidence intervals of high scalp HFO detection rates in EEG recordings with hypsarrhythmia with and without ES relative to IED (multifocal, non‐multifocal).

	Proportion (%)	Crude			Adjusted^a^		
Condition		OR	95% CI	*P*‐value	OR	95% CI	*P*‐value
IED (multifocal, non‐multifocal)	28.6		1.00			1.00	
Hypsarrhythmia without ES	81.8	7.38	1.75–31.06	**0.006**	8.38	1.94–36.20	**0.004**
Hypsarrhythmia with ES	92.3	17.132	3.47–84.70	**<0.001**	24.66	4.86–125.10	**<0.001**

*Note*: The *P*‐values and ORs (95% CIs) were calculated using a GEE model. The *P*‐values < 0.05 are shown in bold and considered statistically significant.

Abbreviations: CI, confidence interval; ES, epileptic spasms; GEE, generalized estimating equation; HFO, high‐frequency oscillation; IED, interictal epileptiform discharge; OR, odds ratio.

^a^ Adjusted for age at the time of the EEG recording.

### 3.4 ROC analysis of scalp HFO detection rates and spike detection rates for reflecting ES activity

Figure 3 shows the ROC analysis based on GLMM‐derived predicted probabilities comparing the ability of scalp HFO detection rates and spike detection rates to discriminate epileptic spasm activity. The scalp HFO detection rate achieved an AUC of 0.92 [95% CI: 0.82–1.00], with a sensitivity of 100.0% and a specificity of 77.4%. The spike detection rate achieved an AUC of 0.86 [0.77–0.99], with a sensitivity of 100.0% and a specificity of 66.0%.

**FIGURE 3:**
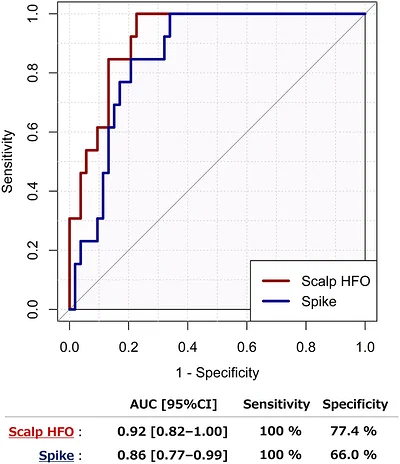
ROC curves for scalp HFO detection rates and spike detection rates in discriminating the presence of ES. ROC curves were constructed using predicted probabilities derived from generalized linear mixed models to account for repeated EEG recordings within patients. Abbreviations: AUC, area under the ROC curve; CI, confidence interval; ES, epileptic spasms; HFO, high‐frequency oscillation; ROC, receiver operating characteristic.

### 3.5 Comparison of scalp HFO characteristics detected in patients among the three groups: hypsarrhythmia with ES; hypsarrhythmia without ES; and IED

Figure 4 shows a comparison of mean scalp HFO characteristics among the three groups using LMMs. The mean ± SD frequency of the scalp HFOs was significantly lower after age adjustment in both the hypsarrhythmia with ES group (117.6 ± 19.3 Hz) and the hypsarrhythmia without ES group (119.5 ± 16.2 Hz) than in the IED group (126.3 ± 20.4 Hz; with ES, crude *P *= 0.062, age‐adjusted *P *= 0.008; and without ES, crude *P *= 0.065, age‐adjusted *P *= 0.018). No significant difference was observed between hypsarrhythmia with and without ES (crude *P *= 0.908, age‐adjusted *P *= 0.750). For amplitude, no significant differences were observed between the IED group (12.6 ± 5.2 *z*‐score) and either the with ES group (13.4 ± 6.5 *z*‐score; crude *P *= 0.459, age‐adjusted *P *= 0.461) or the without ES group (11.7 ± 4.6 *z*‐score; crude *P *= 0.483, age‐adjusted *P *= 0.526). No significant difference was observed between hypsarrhythmia with and without ES (crude *P *= 0.115, age‐adjusted *P *= 0.133). For duration, the mean duration was significantly longer in the hypsarrhythmia without ES group (33.7 ± 12.0 ms) than in the IED group (33.4 ± 13.1 ms; crude *P *= 0.026, age‐adjusted *P *= 0.031). No significant difference was observed between the IED group and the hypsarrhythmia with ES group (32.5 ± 10.7 ms; crude *P *= 0.134, age‐adjusted *P *= 0.191). No significant difference was observed between hypsarrhythmia with and without ES (crude *P *= 0.355, age‐adjusted *P *= 0.311). For the number of cycles, no significant differences were observed between the IED group (5.0 ± 1.1 cycles) and either the with ES group (4.9 ± 1.0 cycles; crude *P *= 0.399, age‐adjusted *P *= 0.336) or the without ES group (5.0 ± 0.9 cycles; crude *P *= 0.764, age‐adjusted *P *= 0.596). No significant difference was observed between hypsarrhythmia with and without ES (crude *P *= 0.202, age‐adjusted *P *= 0.332).

**FIGURE 4:**
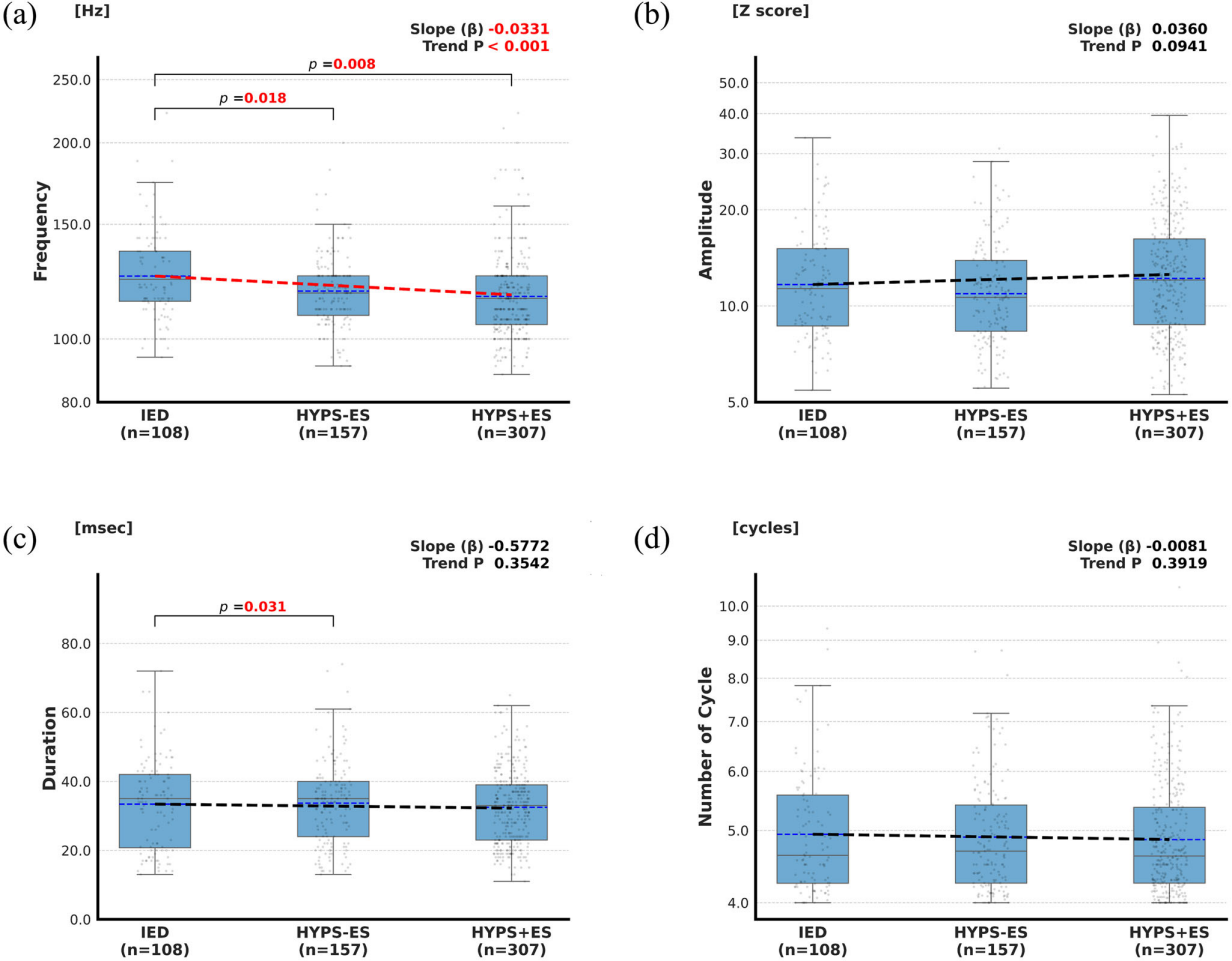
Comparison of scalp HFO characteristics detected in patients among the three groups: hypsarrhythmia with ES; hypsarrhythmia without ES; and IED. Box plots illustrate the comparison of scalp HFO characteristics among the three groups: (a) frequency; (b) amplitude; (c) duration; and (d) number of cycles. *P*‐values and odds ratios (95% confidence intervals) were calculated using generalized estimating equation models. Slope (β) and trend *P*‐values represent the linear association across the three EEG categories (from IED to hypsarrhythmia without ES, to hypsarrhythmia with ES). Abbreviations: ES, epileptic spasms; HFO, high‐frequency oscillation; IED, interictal epileptiform discharge; IESS, infantile epileptic spasms syndrome; LMM, linear mixed‐effects model.

Trend analyses across three categories (from IED to hypsarrhythmia without ES, then to hypsarrhythmia with ES) demonstrated a significant decreasing trend in the frequency [slope (β) = −0.033; trend *P *< 0.001]. For the other HFO characteristics, no significant trends were observed: amplitude [slope (β) = 0.036; trend *P *= 0.094], duration [slope (β) = −0.577; trend *P *= 0.354] and the number of cycles [slope (β) = −0.008; trend *P *= 0.392].

Table 3 shows the results of multiple linear regression analysis for the association of scalp HFO characteristics in the hypsarrhythmia with and without ES groups compared with the IED group using LMMs. The frequency was significantly lower after age adjustment in both hypsarrhythmia with and without ES compared with the IED group (with ES, crude β [95% CI] = −0.094 [−0.194 to 0.006], *P *= 0.065, age‐adjusted β = −0.132 [−0.228 to −0.035], *P *= 0.007; and without ES, crude β = −0.100 [−0.204 to 0.005], *P *= 0.062, age‐adjusted β = −0.118 [−0.215 to −0.020], *P *= 0.018). No significant associations were observed for the amplitude (with ES, crude β = 0.061 [−0.109 to 0.230], *P *= 0.483, age‐adjusted β = 0.059 [−0.123 to 0.240], *P *= 0.526; and without ES, crude β = −0.067 [−0.246 to 0.111], *P *= 0.459, age‐adjusted β = −0.069 [−0.251 to 0.114], *P *= 0.461). Duration was significantly longer in the hypsarrhythmia without ES group (crude β = 0.008 [0.001–0.015], *P *= 0.026; age‐adjusted β = 0.008 [0.001–0.015], *P *= 0.031), whereas no significant association was observed in the hypsarrhythmia with ES group (crude β = 0.005 [−0.002 to 0.012], *P* = 0.134; age‐adjusted β = 0.005 [−0.002 to 0.012], *P* = 0.191). No significant associations were observed for the number of cycles (with ES, crude β = 0.011 [−0.063 to 0.086], *P* = 0.764, age‐adjusted β = 0.019 [−0.050 to 0.087], *P* = 0.596;and without ES, crude β = 0.036 [−0.048 to 0.120], *P* = 0.399, age‐adjusted β = 0.038 [−0.039 to 0.115], *P* = 0.336).

**TABLE 3:** Multiple linear regression analysis of scalp high‐frequency oscillation characteristics in patients with hypsarrhythmia with and without epileptic spasms groups compared with the interictal epileptiform discharge.

Parameter	Crude				Adjusted^a^			
	β	95% CI	Standardized β	*P*‐value	β	95% CI	Standardized β	*P*‐value
**Frequency**								
IED (multifocal, non‐multifocal)	Reference				Reference			
Hypsarrhythmia without ES	−0.100	−0.204 to 0.005	−0.309	0.062	−0.118	−0.215 to −0.020	−0.365	**0.018**
Hypsarrhythmia with ES	−0.094	−0.194 to 0.006	−0.327	0.065	−0.132	−0.228 to −0.035	−0.457	**0.007**
**Amplitude**								
IED (multifocal, non‐multifocal)	Reference				Reference			
Hypsarrhythmia without ES	−0.067	−0.246 to 0.111	−0.081	0.459	−0.069	−0.251 to 0.114	−0.083	0.461
Hypsarrhythmia with ES	0.061	−0.109 to 0.230	0.082	0.483	0.059	−0.123 to 0.240	0.079	0.526
**Duration**								
IED (multifocal, non‐multifocal)	Reference				Reference			
Hypsarrhythmia without ES	0.008	0.001 to 0.015	0.303	**0.026**	0.008	0.001 to 0.015	0.297	**0.031**
Hypsarrhythmia with ES	0.005	−0.002 to 0.012	0.224	0.134	0.005	−0.002 to 0.012	0.205	0.191
**Number of cycles**								
IED (Multifocal, non‐multifocal)	Reference				Reference			
Hypsarrhythmia without ES	0.036	−0.048 to 0.120	0.109	0.399	0.038	−0.039 to 0.115	0.114	0.336
Hypsarrhythmia with ES	0.011	−0.063 to 0.086	0.038	0.764	0.019	−0.050 to 0.087	0.062	0.596

*Note*: Multiple linear regression analysis for the association of scalp HFO characteristics in the hypsarrhythmia with and without ES groups was compared with the IED group using LMMs. *P*‐values < 0.05 are shown in bold and considered statistically significant.

Abbreviations: CI, confidence interval; ES, epileptic spasms; HFO, high‐frequency oscillation; LLM, linear mixed‐effects model.

^a^ Adjusted for age at the time of the EEG recording.

Among the scalp HFO characteristics evaluated, frequency showed the strongest association with the hypsarrhythmia groups, as demonstrated by the largest effect sizes in the standardized models (adjusted standardize β, −0.457 for hypsarrhythmia with ES and −0.365 for hypsarrhythmia without ES).

## 4 DISCUSSION

In this study, we investigated the association among scalp HFOs, hypsarrhythmia and ES in paediatric patients with IESS using scalp EEG recordings. Our findings demonstrated that scalp HFOs were detected most frequently in EEG recordings with hypsarrhythmia, particularly those with ES, and that detection rates were significantly higher in hypsarrhythmia than in IED groups. ROC analysis further demonstrated that scalp HFO detection rates accurately discriminated the presence of ES, supporting their utility as a quantitative marker of ES activity. Furthermore, patients with hypsarrhythmia and ES exhibited distinct morphological features, characterized by lower‐frequency HFOs compared with patients with IED, with the lowest HFO frequencies being observed in patients with hypsarrhythmia accompanied by ES. Taken together, these findings highlight the potential of scalp HFO profiles as clinically meaningful markers in IESS.

Previous studies have reported that scalp HFOs occur in various epilepsy syndromes, including IESS (Fan et al., 2021; Frauscher et al., 2017; Kobayashi et al., 2015, 2016; Maeda, Tsuboi, Hosoda et al., 2025). Consistent with these findings, the present study confirmed the presence of scalp HFOs in patients with IESS. Importantly, scalp HFO detection rates were markedly higher in patients with hypsarrhythmia than in those with IED and were particularly elevated in patients with hypsarrhythmia accompanied by ES. A previous study also reported an association between hypsarrhythmia and scalp HFOs in patients with IESS; however, that investigation had notable methodological limitations (Kobayashi et al., 2015). First, scalp EEG data were acquired at a sampling frequency of 500 Hz. HFOs (ripples) are defined as transient bursts of EEG activity in the 80–250 Hz range. Therefore, EEG data recorded at 500 Hz might have failed to capture HFOs adequately, particularly those occurring near 250 Hz. Second, HFO detection was based on only 60 s of scalp EEG recordings per patient, which might have been insufficient to capture the full extent of HFO activity. Third, the statistical analyses relied solely on univariate methods without adjustment for confounding factors such as age at the time of the EEG recording. Indeed, scalp HFOs have been reported to be associated with age (Windhager et al., 2021), suggesting that potential confounding effects could not be excluded. In contrast, in the present study we overcame these methodological shortcomings systematically. First, we analysed the ripple band (80–250 Hz) using 600 s of scalp EEG data recorded at a sampling frequency of 1000 Hz, which allowed us to capture HFOs across the entire frequency range, including those near 250 Hz that might have been missed in prior work. Second, by extending the recording duration 10‐fold compared with earlier studies, we ensured a more comprehensive characterization of HFO activity. Third, we adjusted for age in the statistical analyses, thereby minimizing the influence of this important confounding factor. Taken together, these methodological advances not only strengthen the validity of our results, but also enhance their clinical applicability, providing a more reliable foundation for future research and practice.

Beyond detection rates, the morphological characteristics of scalp HFOs provide important additional insights. Previous studies have demonstrated that HFO detection rates can increase even in physiological brain regions and vary by anatomical location (Frauscher et al., 2018; Guragain et al., 2018). To distinguish pathological from physiological HFOs, classification has traditionally relied on detection rate thresholds, supplemented by analyses of morphological characteristics (Alkawadri et al., 2014; Bruder et al., 2017; Burnos et al., 2016; Cimbalnik et al., 2018; Matsumoto et al., 2013; Roehri et al., 2018). In the present study, we quantitatively analysed four key morphological characteristics of scalp HFOs (frequency, amplitude, duration and number of cycles) in patients with IESS. Importantly, our analysis focused on all detected scalp HFOs and compared their characteristics among the IED group, hypsarrhythmia without ES group and hypsarrhythmia with ES group. The results demonstrated that both hypsarrhythmia groups exhibited HFOs characterized by lower frequencies compared with the IED group, while the hypsarrhythmia with ES group showed the lowest‐frequency HFOs among the three groups. To our knowledge, few studies have examined scalp HFO morphology in IESS or explored its relationship with ES status. The present findings revealed significant differences in frequency between these groups, suggesting that morphological features might provide clinically relevant information that extends beyond detection rates alone.

An important point to emphasize is that all analyses were conducted on interictal EEG segments. Although routine scalp EEG recordings can occasionally capture clinical seizures, the consistent acquisition of ictal EEG typically requires long‐term video‐EEG monitoring (Tatum et al., 2022). In contrast, interictal HFOs can be detected reliably in standard EEG recordings, offering a practical advantage for clinical application. By focusing on interictal HFO morphology, the present study highlights the potential to infer ES‐related epileptic activity without the necessity for ictal recordings. In contrast, scalp HFO detection can be performed semi‐automatically, providing a more standardized and reproducible approach (Cserpan et al., 2023; Nariai et al., 2020). Importantly, interictal hypsarrhythmia alone cannot adequately reflect the frequency or activity of ES, whereas our findings suggest that interictal scalp HFOs might reflect ES‐related epileptic activity even in the absence of ictal recordings. These findings suggest that detection rate and frequency characteristics of scalp HFOs might serve as a complementary, objective indicator of hypsarrhythmia and ES. Taken together, investigation of the detection rates and morphological features of interictal scalp HFOs constitutes a robust and objective biomarker, thereby offering critical leverage for assessing ictal states and hypsarrhythmia in IESS.

Scalp HFO analysis should not be considered a replacement for conventional EEG analysis but instead is likely to serve as a complementary quantitative biomarker. Conventional EEG approaches, including visual spike assessment and power spectral density analysis, are well established for evaluating epileptiform discharges and oscillatory activity within conventional frequency bands (Liu et al., 2023; Specchio et al., 2022). However, transient high‐frequency oscillatory events (>80 Hz) are not directly quantified by these methods. In contrast, scalp HFO analysis enables semi‐automated detection and objective quantification of these events using reproducible metrics, including detection rate, frequency, amplitude, duration and number of cycles (Zhou et al., 2021). Previous studies have demonstrated that semi‐automated scalp HFO detection is both practical and sufficiently efficient for objective quantitative analysis (Nariai et al., 2020). Furthermore, previous studies evaluating automated scalp HFO analysis have shown that prediction models without visual validation achieved performance comparable to models incorporating visual validation (Nariai et al., 2020), suggesting that fully automated approaches might be feasible in the future. Although visual validation of automatically detected HFOs might introduce some operator dependence in the present study, this limitation might be mitigated as automated detection algorithms continue to improve. In the present study, spike detection rates were calculated using Persyst 14, a commercially available automated spike detection system that has previously been shown to perform comparably to expert human reviewers in large validation studies (Joshi et al., 2018; Scheuer et al., 2017). Comparison of these spike detection rates with scalp HFO detection rates demonstrated that scalp HFOs provided at least comparable performance for quantitatively reflecting epileptic spasm activity. Taken together, these findings suggest that scalp HFO analysis might provide complementary information beyond conventional EEG measures and might be useful as an objective quantitative marker of ES activity.

Regarding the potential mechanistic implications of scalp HFOs in IESS, the increased detection rate and altered frequency characteristics observed in the present study might reflect differences in the underlying state of epileptogenic networks. Although scalp HFOs do not reveal cellular mechanisms directly, prior experimental and intracranial studies suggest several possible interpretations. Bragin et al. (2011) proposed that pathological HFOs might arise from disturbed interactions between excitatory pyramidal neurons and inhibitory interneurons, resulting in abnormally synchronized neuronal firing. In this context, the increased occurrence of scalp HFOs observed in hypsarrhythmia, particularly in cases accompanied by ES, might be consistent with enhanced network excitability and hypersynchronous neuronal activity. In addition, Matsumoto et al. (2013) suggested that pathological HFOs often concentrate in relatively lower frequency ranges, and Bikson et al. (2003), using an experimental epilepsy model, showed that the recruitment of multiple small neuronal bursts into larger neuronal assemblies might contribute to the emergence of higher‐frequency oscillations during ictal evolution. From this perspective, the lower‐frequency HFOs observed in hypsarrhythmia with ES might indicate a distinct interictal network state characterized by unstable or incompletely synchronized epileptogenic activity prior to ictal transition. However, such interpretations remain indirect and should be made cautiously, because the mechanisms of scalp‐recorded HFOs in IESS have not been established and might not correspond fully to those inferred from intracranial or experimental data. Nevertheless, our findings raise the possibility that the occurrence and time–frequency characteristics of scalp HFOs might provide useful clues to the dynamic pathophysiology of epileptogenic networks in IESS.

This study has several limitations. First, scalp HFOs were detected using NREM sleep EEG, in which artefacts such as EMG activity are relatively reduced. Therefore, generalizability to awake EEG remains uncertain. Although ‘false scalp HFOs’ attributable to these artefacts were excluded visually in this study, application of the same procedure to awake EEG would be considerably more labour intensive. In addition, because the analysed EEG epochs were obtained retrospectively from clinical recordings, exact matching of sleep stages across recordings and groups was not feasible. Therefore, differences in sleep stage composition might have influenced HFO characteristics and should be considered when interpreting the present findings. Second, the analyses were based on 600 s of NREM sleep EEG per patient rather than the entire recording. Although previous studies suggest that 600 s is sufficient for reliable HFO detection (Cserpan et al., 2021), it is unclear whether similar results would be obtained using different segments. Third, EEG data were recorded at a sampling frequency of 1000 Hz. In general, EEG measurements require a sampling frequency at least three times greater than the maximum frequency of interest (Halford et al., 2016). Although this captures most HFOs within the 80–250 Hz range, higher sampling rates could improve detection accuracy; thus, future studies should validate these findings using higher frequencies. Fourth, the relatively small sample size (*n* = 66) limited the ability to adjust for multiple confounders. With a larger cohort, it would have been possible to perform more comprehensive multivariable analyses to account for additional confounding factors beyond age. Such limitations are difficult to avoid when investigating rare conditions, such as IESS, which has an estimated incidence of ∼30 per 100 000 live births (Zuberi et al., 2022). However, the potential impact of this limitation on the generalizability of our findings should be considered carefully. To validate and expand upon these results, future studies should aim to include larger patient populations, and multicentre collaborations will be essential to achieve this. Fifth, a methodological consideration of the present study is the handling of HFOs detected simultaneously across multiple EEG channels. In the primary analysis, these HFOs were considered non‐independent observations, and only the event with the largest amplitude was retained to minimize the potential inflation of independent observations. However, given the multifocal nature of IESS, together with previous evidence suggesting that pathological HFOs might arise from abnormal synchronization of neuronal firing caused by disrupted interactions between excitatory pyramidal neurons and inhibitory interneurons, it cannot be completely excluded that some HFOs detected simultaneously across different EEG channels might represent distinct events rather than a single event observed across multiple channels (Bragin et al., 2011; Zuberi et al., 2022). Therefore, the results obtained when concurrently detected HFOs were treated as separate events are also provided in Appendix Tables A2 and A3 and Figures A2, A3, A4. Finally, although all included patients had the initial onset of epileptic spasms within the diagnostic age window for IESS, some EEG recordings were obtained at later stages of the disease course, including cases with clinical relapse, persistent disease activity or evolution into Lennox–Gastaut syndrome. This might have introduced clinical heterogeneity into the cohort. To assess the impact of this heterogeneity, we performed an additional subgroup analysis including only EEG recordings obtained within the diagnostic age window for IESS (≤24 months of age) and compared scalp HFO detection rates among the same four study groups. Although the smaller sample size reduced the statistical significance, the overall pattern of the results was similar to that of the primary analysis. These supplementary results are provided in Appendix Figure A5. Future multicentre collaborations with larger, more homogeneous patient populations will be essential to validate these findings across specific stages of the IESS clinical spectrum.

## 5 CONCLUSION

In summary, in the present study, scalp HFOs were detected at significantly higher rates in EEG recordings with hypsarrhythmia than in those with IED, and this elevated risk persisted after adjustment for age. In addition, scalp HFO detection rates demonstrated good discrimination of ES activity, supporting their potential as a quantitative marker of ictal states. Moreover, patients with hypsarrhythmia showed distinct morphological features, characterized by lower‐frequency HFOs compared with patients with IED, with the lowest HFO frequencies being observed in patients with hypsarrhythmia accompanied by ES. Although the detection rates of scalp HFOs and morphological features are not yet ready for direct clinical application as standalone tools for hypsarrhythmia or assessment of ictal states, our findings support their promise as complementary biomarkers. Future large‐scale, longitudinal studies are needed to validate our results and establish the clinical utility of scalp HFOs in the diagnosis and therapeutic management of IESS.

## AUTHOR CONTRIBUTIONS

Conception or design of the work: Shunta Yamaguchi and Keisuke Maeda. Acquisition, analysis or interpretation of data for the work: Shunta Yamaguchi, Keisuke Maeda, Naoko Ishihara, Gen Furukawa, Himari Tsuboi, Keisuke Osakabe, Keiko Sugimoto and Naohiro Ichino. Drafting the work or revising it critically for important intellectual content: Shunta Yamaguchi, Keisuke Maeda, Naoko Ishihara, Gen Furukawa, Himari Tsuboi, Keisuke Osakabe, Keiko Sugimoto and Naohiro Ichino. All authors have approved the final version of the manuscript; agree to be accountable for all aspects of the work in ensuring that questions related to the accuracy or integrity of any part of the work are appropriately investigated and resolved; and confirm that all persons designated as authors qualify for authorship, and all those who qualify for authorship are listed.

## CONFLICT OF INTEREST

None declared.

## GENERATIVE AI STATEMENT

We confirm that no generative artificial intelligence tools were used in the preparation of this manuscript, including data analysis, interpretation, manuscript drafting or figure generation.

## Figures and Tables

**FIGURE A1:**
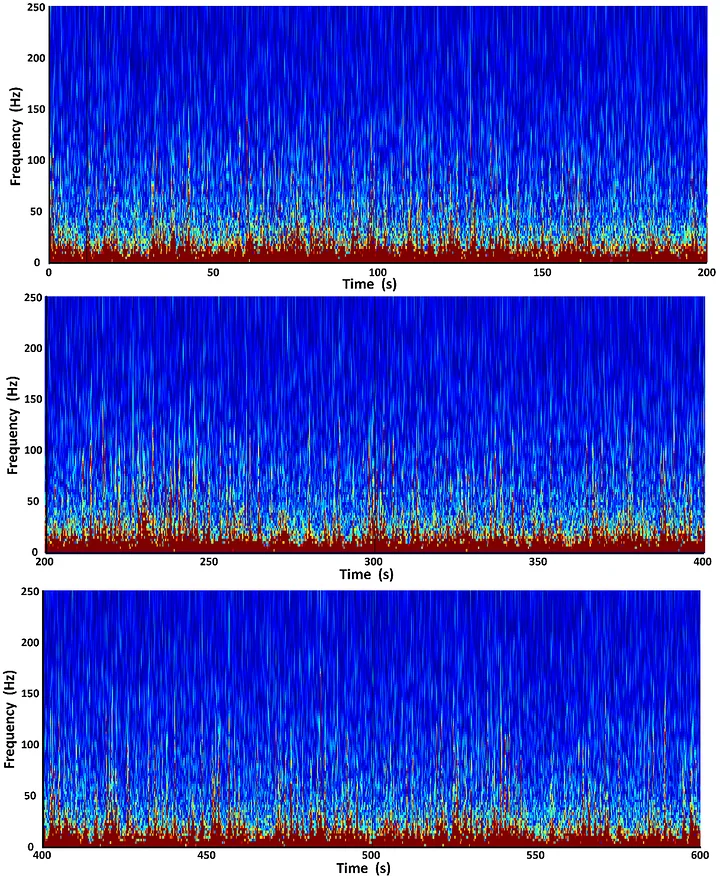
Representative time–frequency spectrogram of a 600 s EEG recording from a patient with hypsarrhythmia with epileptic spasms.

**FIGURE A2:**
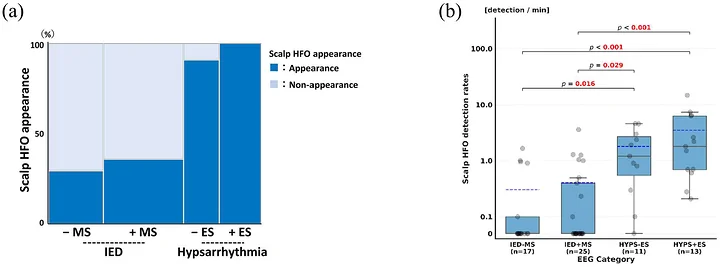
Comparison of scalp HFOs among the four groups: hypsarrhythmia with ES; hypsarrhythmia without ES; multifocal IED; and non‐multifocal IED. (a) Mosaic plot showing the proportion of EEG recordings with at least one scalp HFO detected in each group. (b) Box plot comparing scalp HFO detection rates among the four groups. Each box plot shows the median (bold horizontal line), mean (dotted horizontal line), interquartile range (box) and non‐outlier range (whiskers). *P*‐values were calculated using a linear mixed‐effects model. Abbreviations: ES, epileptic spasms; HFO, high‐frequency oscillation; IED, interictal epileptiform discharge; IESS, infantile epileptic spasms syndrome; MS, multifocal spike.

**FIGURE A3:**
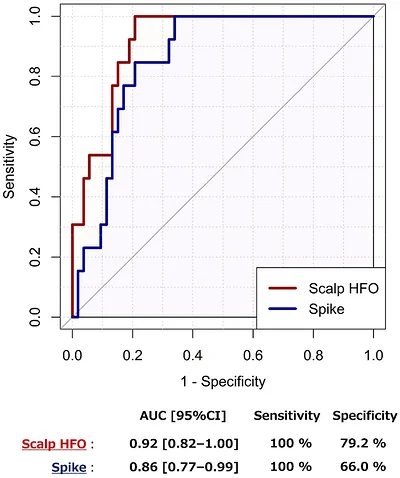
ROC curves for scalp HFO detection rates and spike detection rates in discriminating the presence of ES. ROC curves were constructed using predicted probabilities derived from generalized linear mixed models to account for repeated EEG recordings within patients. Abbreviations: AUC, area under the ROC curve; CI, confidence interval; ES, epileptic spasms; HFO, high‐frequency oscillation; ROC, receiver operating characteristic.

**FIGURE A4:**
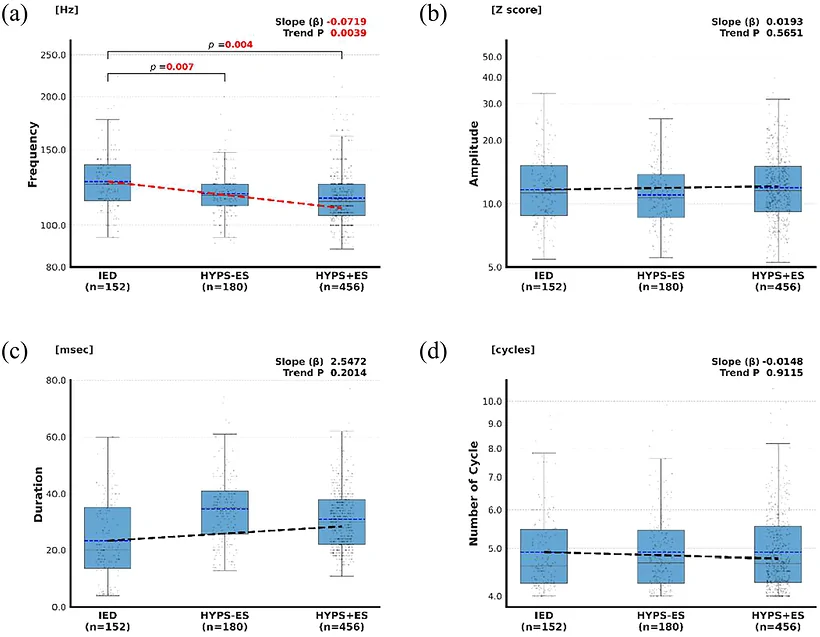
Comparison of scalp HFO characteristics detected in patients among the three groups: hypsarrhythmia with ES; hypsarrhythmia without ES; and IED. Box plots illustrate the comparison of scalp HFO characteristics among the three groups (a) frequency; (b) amplitude; (c) duration; and (d) number of cycles. *P*‐values and odds ratios (95% confidence intervals) were calculated using generalized estimating equation models. Slope (β) and trend *P*‐values represent the linear association across the three EEG categories (from IED to hypsarrhythmia without ES, to hypsarrhythmia with ES). Abbreviations: ES, epileptic spasms; HFO, high‐frequency oscillation; IED, interictal epileptiform discharge; LMM; linear mixed‐effects model.

**FIGURE A5:**
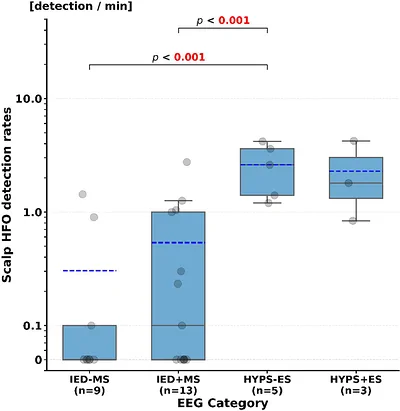
Comparison of scalp HFOs among the four groups including only EEG recordings obtained within the diagnostic age window for IESS (≤24 months of age): hypsarrhythmia with ES; hypsarrhythmia without ES; multifocal IED; and non‐multifocal IED. Box plot comparing scalp HFO detection rates among the four groups. Each box plot shows the median (bold horizontal line), mean (dotted horizontal line), interquartile range (box) and non‐outlier range (whiskers). The *P*‐values were calculated using a linear mixed‐effects model. Abbreviations: ES, epileptic spasms; HFO, high‐frequency oscillation; IED, interictal epileptiform discharge; IESS, infantile epileptic spasms syndrome; MS, multifocal spike.

## Data Availability

The data that support the findings of this study are available on request from the corresponding author.

## 1

**TABLE A1:** Detailed information on all EEG recordings.

EEG no.	Patient no.	Date of EEG recording	EEG category	Number of EEG epochs (raw data)	Number of EEG epochs (selected data)	Number of EEG channels (raw data)	Number of EEG channels (selected data)	Sleep stage N1 (raw data)	Sleep stage N2 (raw data)	Sleep stage N3 (raw data)	Sleep stage R (raw data)	Sleep stage N1 (selected data)	Sleep stage N2 (selected data)	Sleep stage N3 (selected data)	Sleep stage R (selected data)	Ictal EEG (number of captured ictal)	Ictal EEG (number of captured ictal)	Ictal rates (/60 EEG epochs)	Ictal rates (/60 EEG epochs)	Interval [epochs] (ictal to selected epochs)
1	1	24 August 2022	Hypsarrhythmia without ES	241	60	19	19	+	+	―	―	+	+	―	―	―	―	―	―	―
2	1	19 December 2022	Hypsarrhythmia with ES	205	60	19	19	+	+	―	―	+	+	―	―	―	―	―	―	―
3	1	22 March 2023	Hypsarrhythmia with ES	228	60	19	19	+	+	―	―	+	+	―	―	―	―	―	―	―
4	2	18 March 2022	Multifocal IED	181	60	19	19	+	+	―	―	+	+	―	―	―	―	―	―	―
5	2	14 March 2023	Multifocal IED	186	60	19	19	+	+	―	―	+	+	―	―	―	―	―	―	―
6	2	12 March 2024	Hypsarrhythmia with ES	198	60	19	19	+	+	―	―	+	+	―	―	―	―	―	―	―
7	3	13 March 2018	Multifocal IED	372	46	19	19	+	―	―	―	+	―	―	―	―	―	―	―	―
8	4	1 March 2022	Multifocal IED	230	48	19	19	+	+	―	―	+	+	―	―	―	―	―	―	―
9	4	8 March 2022	Non―multifocal IED	347	60	19	19	+	+	―	―	+	+	―	―	―	―	―	―	―
10	5	1 May 2023	Non―multifocal IED	314	61	19	19	+	+	+	―	+	+	+	―	―	―	―	―	―
11	6	10 May 2023	Non―multifocal IED	186	62	19	19	+	―	―	―	+	―	―	―	―	―	―	―	―
12	7	21 May 2020	Hypsarrhythmia without ES	504	60	19	19	―	+	+	―	―	+	+	―	―	―	―	―	―
13	7	27 May 2020	Hypsarrhythmia without ES	196	60	19	19	+	+	+	―	+	+	+	―	―	―	―	―	―
14	8	3 March 2022	Multifocal IED	245	43	19	19	+	+	―	―	+	+	―	―	―	―	―	―	―
15	9	12 January 2022	Non‐multifocal IED	247	60	19	19	+	+	―	―	+	+	―	―	―	―	―	―	―
16	9	27 April 2022	Non‐multifocal IED	183	57	19	19	+	+	―	―	+	+	―	―	―	―	―	―	―
17	9	20 July 2022	Non‐multifocal IED	210	66	19	19	+	+	―	―	+	+	―	―	―	―	―	―	―
18	9	2 November 2022	Non‐multifocal IED	223	60	19	19	+	+	―	―	+	+	―	―	―	―	―	―	―
19	9	1 February 2023	Multifocal IED	179	60	19	19	+	+	―	―	+	+	―	―	―	―	―	―	―
20	9	10 May 2023	Multifocal IED	187	39	19	19	+	+	―	―	+	+	―	―	―	―	―	―	―
21	9	15 November 2023	Multifocal IED	181	52	19	19	+	+	―	―	+	+	―	―	―	―	―	―	―
22	9	22 May 2024	Multifocal IED	184	60	19	19	+	+	―	―	+	+	―	―	―	―	―	―	―
23	10	14 June 2022	Multifocal IED	196	60	19	19	+	+	―	―	+	+	―	―	―	―	―	―	―
24	10	25 November 2022	Hypsarrhythmia without ES	261	60	19	19	+*	+*	―	―	+*	+*	―	―	―	―	―	―	―
25	10	28 April 2023	Hypsarrhythmia without ES	338	60	19	19	―	+*	―	―	―	+*	―	―	―	―	―	―	―
26	10	21 August 2023	Hypsarrhythmia with ES	712	60	19	19	+*	+*	―	―	+*	+*	―	―	3	3	0.25	0.25	91
27	11	5 March 2023	Multifocal IED	199	60	19	19	+	+	―	―	+	+	―	―	―	―	―	―	―
28	11	28 June 2023	Hypsarrhythmia without ES	190	50	19	19	+*	+*	―	―	+*	+*	―	―	―	―	―	―	―
29	11	19 August 2023	Hypsarrhythmia without ES	190	60	19	19	+*	+*	―	―	+*	+*	―	―	―	―	―	―	―
30	11	25 September 2023	Hypsarrhythmia with ES	213	43	19	19	+*	+*	―	―	+*	+*	―	―	―	―	―	―	―
31	11	14 November 2023	Hypsarrhythmia with ES	201	60	19	19	+*	+*	―	―	+*	+*	―	―	―	―	―	―	―
32	11	17 January 2024	Multifocal IED	186	60	19	19	+	+	―	―	+	+	―	―	―	―	―	―	―
33	11	9 April 2024	Hypsarrhythmia with ES	223	60	19	19	+*	+*	+*	―	+*	+*	+*	―	―	―	―	―	―
34	12	24 April 2023	Multifocal IED	210	57	19	19	+	+	―	―	+	+	―	―	―	―	―	―	―
35	12	1 May 2023	Non‐multifocal IED	191	55	19	19	+	+	―	―	+	+	―	―	―	―	―	―	―
36	12	9 May 2023	Multifocal IED	254	60	19	19	+	+	―	―	+	+	―	―	―	―	―	―	―
37	12	22 May 2023	Non‐multifocal IED	227	60	19	19	+	+	―	―	+	+	―	―	―	―	―	―	―
38	12	11 July 2023	Non‐multifocal IED	182	60	19	19	―	+	―	―	―	+	―	―	―	―	―	―	―
39	12	28 November 2023	Non‐multifocal IED	280	60	19	19	+	+	―	―	+	+	―	―	―	―	―	―	―
40	12	8 March 20248	Non‐multifocal IED	184	60	19	19	―	+	―	―	―	+	―	―	―	―	―	―	―
41	13	7 January 2023	Hypsarrhythmia without ES	247	60	19	19	+	+	+	―	+	+	+	―	―	―	―	―	―
42	13	17 Febrary 2023	Non‐multifocal IED	228	60	19	19	―	+	―	―	―	+	―	―	―	―	―	―	―
43	13	14 April 2023	Multifocal IED	182	52	19	19	+	+	+	―	+	+	+	―	―	―	―	―	―
44	13	18 October 2023	Non‐multifocal IED	198	60	19	19	+	+	+	―	+	+	+	―	―	―	―	―	―
45	14	15 September 2022	Hypsarrhythmia with ES	5760	61	19	19	+	+	+	―	+	+	+	―	30	>30	0.31	0.31	700
46	14	22 September 2022	Hypsarrhythmia with ES	452	60	19	19	+	+	―	―	+	+	―	―	―	―	―	―	―
47	15	8 February 2023	Multifocal IED	298	60	19	19	+	+	+	―	+	+	+	―	―	―	―	―	―
48	15	22 February 2023	Non‐multifocal IED	235	56	19	19	+	+	―	―	+	+	―	―	―	―	―	―	―
49	15	31 May 2023	Multifocal IED	183	60	19	19	―	+	―	―	―	+	―	―	―	―	―	―	―
50	15	19 June 2023	Multifocal IED	213	60	19	19	+	+	―	―	+	+	―	―	―	―	―	―	―
51	15	31 July 2023	Multifocal IED	198	60	19	19	+	+	―	―	+	+	―	―	―	―	―	―	―
52	15	19 September 2023	Multifocal IED	197	60	19	19	+	+	―	―	+	+	―	―	―	―	―	―	―
53	15	9 November 2023	Multifocal IED	192	60	19	19	+	+	―	―	+	+	―	―	―	―	―	―	―
54	15	7 February 2024	Non―multifocal IED	186	60	19	19	+	+	―	―	+	+	―	―	―	―	―	―	―
55	16	18 October 2023	Non―multifocal IED	250	60	19	19	+	+	―	―	+	+	―	―	―	―	―	―	―
56	17	27 September 2021	Hypsarrhythmia without ES	586	59	19	19	+	―	―	―	+	―	―	―	―	―	―	―	―
57	17	19 January 2022	Hypsarrhythmia without ES	229	43	19	19	+*	+*	―	―	+*	+*	―	―	―	―	―	―	―
58	17	26 February 2022	Hypsarrhythmia with ES	5827	59	19	19	+*	+*	+*	―	+*	+*	+*	―	―	―	―	―	―
59	18	9 June 2021	Hypsarrhythmia with ES	180	29	19	19	+*	+*	―	―	+*	+*	―	―	4	4	1.33	1.33	60
60	18	7 July 2021	Hypsarrhythmia with ES	191	43	19	19	+*	+*	―	―	+*	+*	―	―	3	3	0.94	0.94	21
61	18	15 June 2022	Hypsarrhythmia without ES	259	60	19	19	+*	+*	―	―	+*	+*	―	―	―	―	―	―	―
62	18	10 May 2023	Multifocal IED	184	31	19	19	+	+	―	―	+	+	―	―	―	―	―	―	―
63	19	4 September 2018	Hypsarrhythmia with ES	497	60	19	19	+*	+*	―	―	+*	+*	―	―	30	>30	3.62	>3.62	172
64	20	11 April 2018	Multifocal IED	362	60	19	19	+	+	―	―	+	+	―	―	―	―	―	―	―
65	20	10 July 2019	Multifocal IED	509	57	19	19	+	+	―	―	+	+	―	―	―	―	―	―	―
66	20	8 January 2020	Multifocal IED	189	45	19	19	+	+	―	―	+	+	―	―	―	―	―	―	―

Abbreviations: ES, epileptic spasms; IED, interictal epileptiform discharge.

* Sleep stage could not be determined with sufficient confidence because physiological sleep features were absent or obscured by severe hypsarrhythmia. Sleep staging was performed by a board‐certified epileptologist.

**TABLE A2:** Odds ratios and 95% confidence intervals of high scalp high‐frequency oscillation detection rates in EEG recordings with hypsarrhythmia with and without epileptic spasms relative to interictal epileptiform discharge (multifocal, non‐multifocal).

	Proportion (%)	Crude			Adjusted^a^		
Condition		OR	95% CI	*P*‐value	OR	95% CI	*P*‐value
IED (multifocal, non‐multifocal)	32.0		1.00			1.00	
Hypsarrhythmia without ES	81.8	11.25	2.50–50.62	**0.002**	13.52	2.99–61.06	**<0.001**
Hypsarrhythmia with ES	92.3	30.00	4.25–211.93	**<0.001**	52.52	5.16–534.63	**<0.001**

*Note*: *P*‐values and ORs (95% CIs) were calculated using a generalized estimating equation model. *P*‐values < 0.05 are shown in bold and considered statistically significant.

Abbreviations: CI, confidence interval; ES, epileptic spasms; HFO, high‐frequency oscillation; IED, interictal epileptiform discharge; OR, odds ratio.

^a^ Adjusted for age at the time of the EEG recording.

**TABLE A3:** Multiple linear regression analysis of scalp high‐frequency oscillation characteristics in patients with hypsarrhythmia with and without epileptic spasms groups compared with the interictal epileptiform discharge.

Parameter	Crude				Adjusted^a^			
	β	95% CI	Standardized β	*P*‐value	β	95% CI	Standardized β	*P*‐value
**Frequency**								
IED (Multifocal, Non‐multifocal)	Reference				Reference			
Hypsarrhythmia without ES	−0.108	−0.212–−0.004	−0.738	**0.042**	−0.121	−0.221–−0.020	−0.825	**0.018**
Hypsarrhythmia with ES	−0.101	−0.197–−0.006	−0.692	**0.038**	−0.139	−0.237–−0.041	−0.947	**0.006**
**Amplitude**								
IED (multifocal, non‐multifocal)	Reference				Reference			
Hypsarrhythmia without ES	−0.079	−0.233 to 0.076	−0.210	0.319	−0.079	−0.237 to 0.079	−0.211	0.329
Hypsarrhythmia with ES	0.039	−0.102 to 0.180	0.105	0.587	0.042	−0.110 to 0.194	0.112	0.589
**Duration**								
IED (multifocal, non‐multifocal)	Reference				Reference			
Hypsarrhythmia without ES	5.869	−2.034 to 13.772	0.467	0.146	5.884	−2.182 to 13.949	0.469	0.153
Hypsarrhythmia with ES	4.680	−2.602 to 11.962	0.373	0.208	4.720	−3.153 to 12.592	0.376	0.240
**Number of cycles**								
IED (multifocal, non‐multifocal)	Reference				Reference			
Hypsarrhythmia without ES	−0.003	−0.053 to 0.048	−0.016	0.910	0.000	−0.048 to 0.048	−0.001	0.995
Hypsarrhythmia with ES	−0.010	−0.055 to 0.036	−0.053	0.682	0.003	−0.042 to 0.048	0.016	0.900

*Note*: Multiple linear regression analysis for the association of scalp HFO characteristics in the hypsarrhythmia with and without ES groups compared with the IED group using linear mixed‐effects models. *P*‐values < 0.05 are shown in bold and considered statistically significant.

Abbreviations: CI, confidence interval; ES, epileptic spasms; HFO, high‐frequency oscillation.

^a^ Adjusted for age at the time of the EEG recording.
